# The Site/Group Extended Data Format and Tools

**DOI:** 10.1093/gbe/evae011

**Published:** 2024-01-22

**Authors:** Julien Y Dutheil, Diyar Hamidi, Basile Pajot

**Affiliations:** Research Group “Molecular Systems Evolution,” Department of Theoretical Biology, Max Planck Institute for Evolutionary Biology, Plön 24306, Germany; Research Group “Molecular Systems Evolution,” Department of Theoretical Biology, Max Planck Institute for Evolutionary Biology, Plön 24306, Germany; Research Group “Molecular Systems Evolution,” Department of Theoretical Biology, Max Planck Institute for Evolutionary Biology, Plön 24306, Germany

**Keywords:** data structure, comparative sequence analysis, three-dimensional structure, randomization

## Abstract

Comparative sequence analysis permits unraveling the molecular processes underlying gene evolution. Many statistical methods generate candidate positions within genes, such as fast or slowly evolving sites, coevolving groups of residues, sites undergoing positive selection, or changes in evolutionary rates. Understanding the functional causes of these evolutionary patterns requires combining the results of these analyses and mapping them onto molecular structures, a complex task involving distinct coordinate referential systems. To ease this task, we introduce the site/group extended data format, a simple text format to store (groups of) site annotations. We developed a toolset, the SgedTools, which permits site/group extended data file manipulation, creating them from various software outputs and translating coordinates between individual sequences, alignments, and three-dimensional structures. The package also includes a Monte-Carlo procedure to generate random site samples, possibly conditioning on site-specific features. This eases the statistical testing of evolutionary hypotheses, accounting for the structural properties of the encoded molecules.

**Significance**

Testing the predictions generated by comparative sequence analysis requires the integration of functional data of the underlying genes, including the three-dimensional structure of the encoded macro-molecule. This integration involves the mapping of data in different coordinate systems, such as sequence, alignment, and structure. We developed (i) a dedicated file format for the exchange of (groups of) sites in a sequence or sequence alignment together with their annotations and (ii) a series of tools to handle files in this format. These tools automatize coordinate conversion and facilitate the creation of pipelines for the functional and structural analysis of evolutionary predictions.

## Introduction

Evolutionary comparative sequence analysis can unravel information about the evolutionary processes that shape the observed genetic diversity. When applied to gene sequence alignments, dedicated statistical methods detect positions that evolved under a particular evolutionary scenario, such as negative/positive selection or coevolution (Pollock et al. 1999; Yang 2006). Further insights into the functional role of these positions in the molecule and organism can be obtained by mapping them onto the three-dimensional structure of the encoded molecule and assessing their structural properties.

Mapping evolutionary predictions onto three-dimensional structures requires translating positions between three distinct reference systems: alignment positions, individual sequences, and three-dimensional structures. While software that allows the joint visualization of sequence alignments, phylogenies, and protein structures is available (Meng et al. 2006; Waterhouse et al. 2009), it requires manual interaction to visualize the results of evolutionary analyses, restricting their usage to case studies and preventing their use in genomic pipelines. Furthermore, each analysis software outputs results in its distinct format, complicating the development of generic analysis tools.

We designed the site/group extended data (SGED) format to facilitate the cross-analysis of sequence sites and their annotations. We introduce the SgedTools package, which contains utilities to manipulate and analyze SGED files. Lastly, we demonstrate their application on a classic example of positively selected sites in Primates lysozyme sequences.

## The SGED Format

**Fig. 1.**
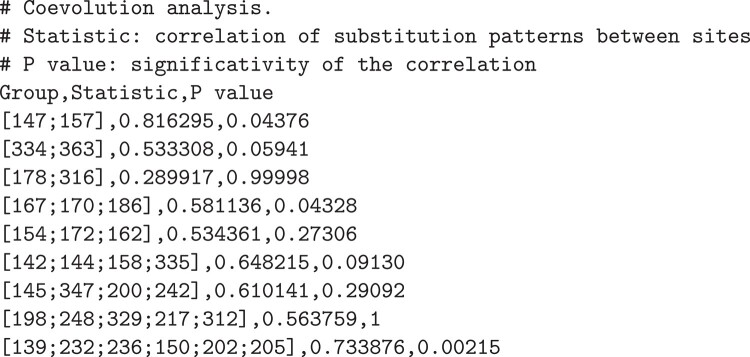
Example of SGED file displaying the results of a coevolution analysis. The first column displays the groups with their alignment coordinates, followed by the coevolution statistics (second column) and the corresponding *P* values (third column). Lines starting with “#” are comments and will be ignored when processing the file.

We propose generalizing the text tabular format to account for site coordinates. The SGED format is based on the widely used comma-separated values (CSVs) and tab-separated values (TSVs) formats, where columns represent variables and rows represent data points—here in the form of (groups of) sites in a sequence or alignment. The SGED file contains one or several columns to store coordinates (e.g. a site’s position in the alignment), with a dedicated syntax: the coordinates are specified within square brackets, and coordinates are separated by semi-columns (see Fig. 1). Other columns represent any measure or statistic for the corresponding groups. Comments and metadata can further be added using the “#” character at the beginning of the line. The SgedTools offers a collection of programs that specifically deal with the coordinates of the groups. They also compute statistics that will be added as columns in the SGED files.

## Generating and Manipulating SGED Files

As SGED files are CSV/TSV files, they can be easily generated and edited, either manually or with dedicated software, such as spreadsheets, R, or the Python package pandas. The format is supported by programs using the Bio++ libraries, outputting various alignment statistics (Guéguen et al. 2013). The SgedTools contains several conversion utilities that generate SGED files from the output of programs for sequence and structure analysis (supplementary table 1, Supplementary Material online). SGED files can be further manipulated by dissociating sites within groups or combining sites into groups according to the content of a column. Finally, the columns of two SGED files can be merged based on the group coordinates.

## Indexing and Coordinate Translation

**Fig. 2.**
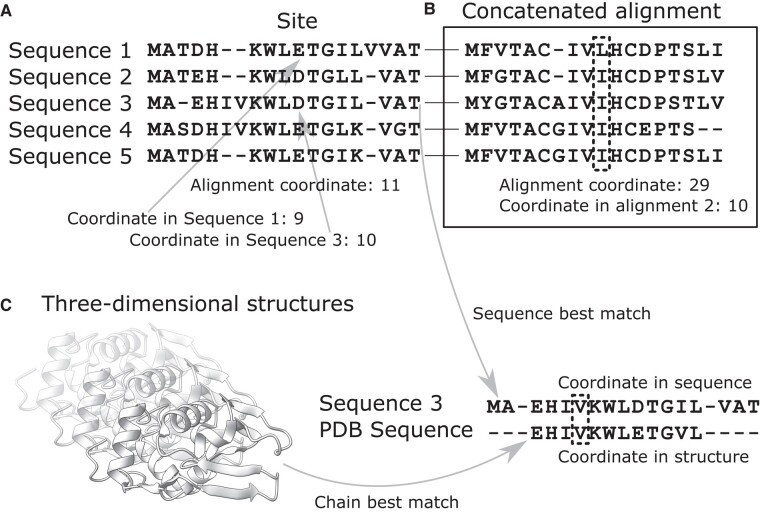
Distinct coordinate systems. A) Sites (= alignment columns) correspond to distinct positions within each aligned sequence. B) When alignments are concatenated, one needs to keep track of the original alignment coordinates in the concatenated alignment. C) To map alignment positions onto a three-dimensional structure, the sequence of each chain must be aligned with each sequence of the alignment to find the best match.

A prerequisite for analyzing candidate positions in a sequence or sequence alignment is the conversion of coordinates to a common reference (supplementary table 1, Supplementary Material online). The most basic conversion is between sequences within an alignment and is easily achieved by indexing each sequence position according to their alignment column (Fig. 2*A*). Another sequence-only conversion task is when sequences or alignments are concatenated, for instance, to reconstruct a joint phylogeny, jointly estimate model parameters on multiple genes, or perform an inter-gene coevolution analysis. Positions in the super-alignment subsequently need to be converted back to the original sequence coordinates for further analysis (Fig. 2*B*).

Coordinates are required to cross results between different analyses, particularly evolutionary analyses (alignment-based) and functional analyses (single-sequence-based). A class of widely used functional analyses involve the three-dimensional structure of the encoded molecule, RNA, or protein. Three-dimensional structures can be obtained experimentally or predicted computationally. In both cases, some data may be missing so that the structure of some part of the sequence could not be obtained. Furthermore, the reference structure may not be from the same individual used for the evolutionary analysis, possibly even from a different species. Mapping candidate sites from evolutionary analyses onto a protein or RNA structure is a challenging task that requires sequence alignment between reference sequences (Fig. 2*C*).

The create-structure-index program from the SgedTools permits the automation of such a task. Using a set of protein data bank (PDB) structures, it aligns each sequence in a sequence alignment with the sequence of every chain in every PDB entry provided as input. Using the best matching pair of sequences, it then creates an alignment-structure index that maps all alignment positions onto the selected three-dimensional structure with minimal data loss. The sequence alignment is done using methods from the BioPython package (Cock et al. 2009). Structure-mapped positions can then be used to extract structural properties.

## Adding Structural Properties

Information about the functional relevance of predicted sites can be obtained by knowledge of their three-dimensional position. Relevant structural characteristics include location in secondary structure motifs, solvent exposure, number of residue contacts, and inter-residue distances. Some information is directly accessible from the three-dimensional structure file; others can be predicted with dedicated software. The structure-infos program uses the BioPython.PDB package (Hamelryck and Manderick 2003) to automatically retrieve structural properties from PDB and mmCIF files, such as secondary structure motives (supplementary table 1, Supplementary Material online). It can also compute three-dimensional distances between sets of residues and can further retrieve information about residues’s relative solvent accessibility (RSA) and depth using the BioPython.PDB parsers for the “Define Secondary Structure of Proteins” (DSSP) (Kabsch and Sander 1983) and Michel Sanner's MSMS (Sanner et al. 1996) programs.

The structure-infos program further features an algorithm computing the number of residue clusters in a group of sites. It first generates the matrix of pairwise distances between all pairs of residues in a group. A hierarchical clustering tree is then computed from the distance matrix, using the nearest linkage algorithm, as implemented in the cluster.hierarchy.single function in the SciPy package (Virtanen et al. 2020). A distance threshold is then used to obtain clusters of residues. To assess the significance of structural statistics, we need to compare their observed values to their expectation under a null model. Such expectations can be derived using randomization procedures.

## Advanced Hypothesis Testing Using Randomization

The randomize-groups program (supplementary table 1, Supplementary Material online) generates random groups from two input SGED files: a first file with the predicted sites to be tested (referred to as “test groups”) and a second file providing the list of sites to sample from (referred to as “input sites”). Typically, the test groups will contain the result of an evolutionary analysis and the input sites the set of all sites that could be included in the analysis. Each site can only be sampled once in each test group, but a site can be sampled multiple times between test groups if several are provided.

randomize-groups can perform a conditional sampling by selecting sites with similar properties to those in the tested group. This is achieved by specifying a conditional variable, provided as a dedicated column in the input sites file. For continuous condition variables, a similarity threshold is set in order to select similar sites. In the case of a skewed distribution, the average of the variable within the selected site set may significantly differ from the value of the tested group, in which case a bias correction is implemented, as described in Chaurasia and Dutheil (2022). In the next section, we demonstrate how the SgedTools can be used to statistically analyze the structural properties of sites detected to evolve under positive selection, using conditional sampling to disentangle the effect of RSA and residue dispersal.

## Application Example: Structural Analysis of Positively Selected Sites

To illustrate the use of the SgedTools, we evaluate the results of the positive selection analysis of Yang and Nielsen (2002). This data set serves as an example for the widely used package phylogenetic analysis by maximum likelihood (PAML) (Yang 2007). The PAML “mlc” output file can be converted to the SGED format using the paml2sged program, keeping only the seven sites with a posterior probability calculated by the empirical Bayesian method and at least equal to 0.7:

sged-paml2sged \

 --paml mlc

 --output lysozymeLarge-possel.sged \

 --method bayesian \

 --threshold 0.7

The resulting file lysozymeLarge-possel.sged has the following content:

Group amino_acid   probability [14]  R   0.859 [21]  R   0.858 [23]  I   0.853 [41]  R   0.71 [50]  R   0.704 [87]  D   0.869 [126] Q   0.71

where the “probability” column indicates the posterior probability of the site to evolve under positive selection. Using the Colobus sequence as a reference, we search the PDB (Berman et al. 2000) for three-dimensional structures of lysozymes. After downloading the ten best matching PDB files, we use the create-structure-index program to align all chains from all structures and find the best alignment, which is used to create a *structure index*:

sged-create-structure-index \

 --pdb "*.pdb" \

 --pdb-format PDB \

 --alignment colobus_aa.fas

 --alignment-format fasta \

 --gap-open -2 \

 --output lysozymeLarge_PdbIndex.txt \

 --exclude-incomplete

We use a gap-opening penalty of −2 to maximize the overlap of the structure with the selected sequence, as they are not from the same species. Incomplete structures are excluded from the comparison. Chain A from the 134L PDB entry was selected as the closest match. We then use the generated index to obtain the coordinates of the positively selected sites in the protein structure:

sged-translate-coords. \

 --sged lysozymeLarge-possel.sged \

 --output lysozymeLarge-possel_PDB.sged \

 --index lysozymeLarge_PdbIndex.txt

 --name PDB

resulting in the SGED file:

Group PDB  amino_acid probability

[14] [A:ARG14]   R 0.859

[21] [A:ARG21]   R 0.858

[23] [A:ILE23]    I 0.853

[41] [A:ARG41]   R 0.71

[50] [A:ARG50]   R 0.704

[87] [A:ASP87]   D 0.869

[126] [A:GLN126]  Q 0.71

**Fig. 3.**
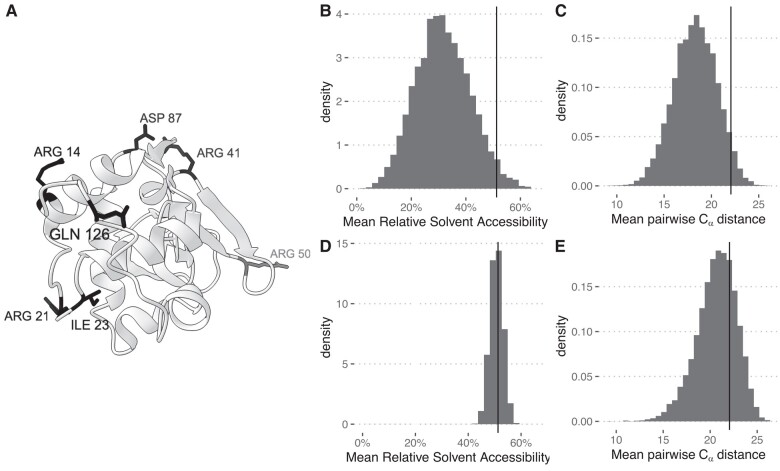
Analysis of positively selected sites in the lysozyme. A) Three-dimensional structure of the human lysozyme (PDB structure 134l). Residues corresponding to sites evolving under a positive selection scenario with a posterior probability higher or equal to 70% are shown in full (labeled residues). B–E) Histograms of distributions over 10,000 random groups. Vertical lines show the corresponding observed values. B and D) Average RSA. C and E) Average pairwise $C_{\alpha }$ distance. B and C) Sampling over all residues in the structure. D and E) Sampling conditioned on the RSA value of each residue.

The translated coordinates can be used to visualize the candidate sites with software like PyMol (Schrödinger 2015) or ChimeraX (Meng et al. 2023; Fig. 3*A*). The positively selected sites are located at the protein’s surface and seem to be spread. We can statistically assess this by measuring the mean pairwise distance between the *α* carbons ($C_{\alpha }$) of the residues and their mean RSA. We first need to create an SGED file where all sites are listed as a single group:

sged-group \

 --sged lysozymeLarge-possel_PDB.sged \

 --group PDB

 --output lysozymeLarge-possel-group.sged

resulting in the following SGED file:

Group

[A:ARG14;A:ARG21;A:ILE23;A:ARG41;A:ARG50;A:ASP87;A:GLN126]

We then compute the structural properties of this group using the structure-info program, using the best matching PDB entry:

sged-structure-infos \

 --sged lysozymeLarge-possel-group.sged \

 --pdb 134l.pdb

 --pdb-format PDB \

 --measure AlphaDist \

 --measure DSSPsum \

 --output lysozymeLarge-possel-group_PDB_infos.sged

The program computes two statistics for the group, the $C_{\alpha }$ distance (argument --measure AlphaDist) and several summary statistics generated by the DSSP program (argument --measure DSSPsum). The resulting mean $C_{\alpha }$ distance is 22.06 Å, and the mean RSA is 51.27%. We then compare these statistics to their null expectation, obtained by sampling groups of sites in the protein structure using the program randomize-groups. We need to provide the list of sites to sample from using the structure-list program:

sged-structure-list \

 --pdb 134l.pdb \

 --pdb-format PDB \

 --output 134l_residues.sged

We generate 10,000 random groups:

sged-randomize-groups \

 --sged-groups lysozymeLarge-possel-group.sged \

 --sged-sites 134l_residues.sged \

 --number-replicates 10000 \

 --output lysozymeLarge-possel-group_random.sged

Finally, we compute the structural properties of the random groups, as it was done for the group of positively selected sites:

sged-structure-infos \

 --sged lysozymeLarge-possel-group_random.sged \

 --pdb 134l.pdb \

 --pdb-format PDB \

 --measure AlphaDist \

 --measure DSSPsum \

 --output lysozymeLarge-possel-group_random_PDB_infos.sged

The two observed statistics appear larger than their random expectation (Fig. 3*B,C*). We compute an upper bound for the *P* value as


(1) $$P\;\mathrm{value}=\frac{|S_{\mathrm{sim}}\geq S_{\mathrm{obs}}|+1}{10,000+1}$$

where $\left|S_{\mathrm{sim}}\geq S_{\mathrm{obs}}\right|$ represents the number of simulated groups with a statistic at least equal to the observed value (one-tail test). This gives 0.0304 for the solvent exposure and 0.0444 for the $C_{\alpha }$ distance, both significant at the 5% level.

These results indicate that the surface exposure of the candidate sites is likely linked to their function. We further ask whether their dispersal is also possibly a signature of their function or whether it is a by-product of their location at the surface of the protein. We perform a *conditional sampling* by sampling exclusive sites with a solvent exposure similar to the candidate sites. For this, we first compute the exposure of every residue of the structure:

sged-structure-infos \

 --sged 134l_residues.sged \

 --pdb 134l.pdb

 --pdb-format PDB \

 --measure DSSP \

 --output 134l_residues_infos.sged

and then condition on the RSA of each site, which is stored in the “Rsa” column of the 134l_residues_infos.sged file:

sged-randomize-groups \

 --sged-groups lysozymeLarge-possel-group.sged \

 --sged-sites 134l_residues_infos.sged \

 --measure Rsa \

 --similarity-threshold 0.2 \

 --number-replicates 10000 \

 --output lysozymeLarge-possel-group_random-rsa.sged

We finally compute the structural characteristics of the random groups:

sged-structure-infos \

 --sged lysozymeLarge-possel-group_random-rsa.sged

 --pdb 134l.pdb \

 --pdb-format PDB

 --measure AlphaDist \

 --measure DSSPsum \

 --output lysozymeLarge-possel-group_random-rsa_PDB.sged

The distribution of the average RSA is now centered on the observed value, showing that the exposure effect is accounted for (Fig. 3*D*). However, the $C_{\alpha }$ distance is no longer significant (Fig. 3*E*), *P* value = 0.2674, indicating that the apparent residues’ dispersal results from a spurious correlation with the RSA.

## Conclusion

We introduced a set of generic tools that permit integrating results from various evolutionary analyses with functional annotations, including three-dimensional structures. This interoperability is made possible by a generic file format for storing position-specific sequence annotations. The format supports annotations for single sites and groups of sites while being simple and flexible. Besides basic data manipulation, the SgedTools implement more complex algorithms for mapping three-dimensional structures to sequence alignments and a conditional sampling of sites for the statistical testing of hypotheses. The tools can be combined as modules to create pipelines for testing functional and structural hypotheses from evolutionary predictions.

## Supplementary Material

evae011_Supplementary_Data

## Data Availability

The SgedTools are distributed under the GNU General Public Licence version 3 (GPL3) and can be downloaded from GitHub.com at https://jydu.github.io/sgedtools/. The package is written in Python (version 3.1 minimum). It makes use of several Python packages: *pandas* for CSV/TSV file reading, manipulating, and writing (The pandas development team 2020), *numpy* and *scipy* for numerical calculations and statistics (Harris et al. 2020; Virtanen et al. 2020), *biopython* for sequence and three-dimensional structures manipulation (Cock et al. 2009). The Bio.PDB module from BioPython provides wrappers for the DSSP (Kabsch and Sander 1983) and MSMS (Sanner et al. 1996) programs, which have to be installed separately. Once the packages are available in the Python environment, each script can be copied and run “as is” without any further installation needed. The package also provides an installation script that can be used to install the programs in a system directory. The programs are run from the command line, using options which are specified using standard short (e.g. -a) or long arguments (e.g. --alignment). The SgedTools package is distributed with detailed example analyses that can serve as templates for developing dedicated pipelines.
