# Supplementary material for: The Site/Group Extended Data Format and Tools
Source: Genome Biol Evol. 2024 Jan 22;16(2):evae011. doi: 10.1093/gbe/evae011 (PMC10849175; doi:10.1093/gbe/evae011)
Supplement: evae011_Supplementary_Data [file evae011_supplementary_data.pdf]

The Site/Group Extended Data format and tools: Supplementary Material

Julien Y. Dutheil[1,*], Diyar Hamidi[1], and Basile Pajot[1]

[1]Research Group "Molecular Systems Evolution", Department of Theoretical Biology, Max Planck Institute for Evolutionary Biology, August-Thienemann-Str. 2, 24306 Plön, Germany
[*]Corresponding author: August-Thienemann-Str. 2, 24306 Plön. dutheil@evolbio.mpg.de

January 8, 2024

Supplementary Table

Supplementary Table 1: List of programs in the SgedTools package.

	Program	Function
Format conversion and file manipulation	`paml2sged`	• Create an SGED file from the output of the `codeml` program from the `PAML` package (sites under positive selection) [Yang, 2007]
	`raser2sged`	• Create an SGED file from the output of `RASER` [Penn et al., 2008] (sites undergoing rate shifts)
	`disembl2sged`	• Create an SGED file from the output of `disembl` [Linding et al., 2003] (intrinsically disordered regions)
	`structure-list`	• Create an SGED from a PDB structure file, listing all residues.
	`get-all-pairs`	• Take all N groups in an SGED file and combine them in pairs, resulting in a file with $\frac{N\cdot(N-1)}{2}$ new groups
	`group`	• Aggregate single sites or groups from an SGED file into (super) groups, possibly according to a given column. For example: $\begin{array}{c} [1], A \\ [2], B \\ [3], A \\ [4], B \end{array} \rightarrow \begin{array}{c} [1;3], A \\ [2;4], B \end{array}$
	`ungroup`	• Unlist all positions within groups. For instance: $[1;2;3], A \rightarrow \begin{array}{c} [1], A \\ [2], A \\ [3], A \end{array}$
	`merge`	• Merge two SGED files based on group coordinates; several join operations are supported
	`sged2deffatr`	• Export data in one column to an attribute file importable in the Chimera/ChimeraX software [Meng et al., 2023]

Supplementary Table 1 (continued): List of programs in the SgedTools package.

	Program	Function
Index and translate coordinates	`concatenate-alignments`	• Concatenate two or more alignments into a super-alignment, with the same input species and one concatenated sequence per species<br>• Create indexes of original positions in the concatenated alignment
	`create-sequence-index`	• Create an index for the positions in a reference sequence in the alignment
	`create-structure-index`	• Match sequences in a sequence alignment with one or more three-dimensional structures<br>• Creates an index of alignment positions in the best-matching structure
	`liftover-index`	• Convert an index A using index B, so that $(A \to B) + (B \to C) = (A \to C)$
	`merge-indexes`	• Combine indexes from distinct, non-overlapping, regions
	`translate-coords`	• Use a previously generated index to convert coordinates of the entries in a SGED file from one reference to another.
Structural properties and statistics	`structure-infos`	• Compute several structural statistics of sites or groups from a three-dimensional structure (including 3D distance, solvent accessibility, secondary structure, and number of clusters)
	`summary`	• Compute summary statistics for all sites in each group based on the properties of each site
	`randomize-groups`	• Generate a random set of groups with characteristics similar to a reference group set. Takes as input a list of single sites with properties and outputs groups with the same size and similar site properties than the reference group set
	`group-test-inclusion`	• Test whether the groups in one file are present in a second file

References

R. Linding, L. J. Jensen, F. Diella, P. Bork, T. J. Gibson, and R. B. Russell. Protein disorder prediction: implications for structural proteomics. *Structure*, 11(11):1453–1459, Nov. 2003. ISSN 0969-2126. doi: 10.1016/j.str.2003.10.002.

E. C. Meng, T. D. Goddard, E. F. Pettersen, G. S. Couch, Z. J. Pearson, J. H. Morris, and T. E. Ferrin. UCSF ChimeraX: Tools for Structure Building and Analysis. *Protein Sci*, page e4792, Sept. 2023. ISSN 1469-896X. doi: 10.1002/pro.4792.

O. Penn, A. Stern, N. D. Rubinstein, J. Dutheil, E. Bacharach, N. Galtier, and T. Pupko. Evolutionary modeling of rate shifts reveals specificity determinants in HIV-1 subtypes. *PLoS Comput. Biol.*, 4(11):e1000214, Nov. 2008. ISSN 1553-7358. doi: 10.1371/journal.pcbi.1000214.

Z. Yang. PAML 4: phylogenetic analysis by maximum likelihood. *Mol. Biol. Evol.*, 24(8): 1586–1591, Aug. 2007. ISSN 0737-4038. doi: 10.1093/molbev/msm088.
